# What you see is not what you get: Observed scale score comparisons misestimate true group differences

**DOI:** 10.3758/s13428-025-02639-w

**Published:** 2025-03-19

**Authors:** Bjarne Schmalbach, Ileana Schmalbach, Jochen Hardt

**Affiliations:** Medical Psychology and Medical Sociology, University Medical Center of the Johannes Gutenberg-University, Mainz, Germany

**Keywords:** Scale score, Factor analysis, Group differences, Structural equation modeling, Measurement error

## Abstract

Social sciences of all kinds are interested in latent variables, their measurement, and how they differ between groups. The present study argues the importance of analyzing mean differences between groups using the latent variable approach. Using an open-access repository of widely applied personality questionnaires (*N* = 999,033), we evaluate the extent to which the commonly used observed sum score is susceptible to measurement error. Our findings show that Cohen’s *d* values based on the observed variance significantly misestimate the true group difference (based on just the factor score variance) in 33 of the 70 studied cases, and by an average of 25.0% (or 0.048 standard deviations). There was no meaningful relationship between the effect size discrepancy and scale reliability as measured by McDonald’s ω. We discuss the implications of these results and outline concrete steps that applied researchers can take to improve their analyses.

**Supplementary Information:**

The online version contains supplementary material available at 10.3758/s13428-025-02639-w.

## Introduction

Item-based questionnaires are the backbone of many basic and applied research strands dealing with latent constructs, i.e., such concepts that cannot be directly observed or measured. Social and personality research in particular is known to rely heavily on self-report measures that ask for participants’ subjective evaluations of any given topic (Paulhus & Vazire, 2007). These individual evaluations are subsequently aggregated into a scale score and interpreted as an approximation of the latent construct in question. As such, these measures are absolutely indispensable for a vast array of social sciences (Flake et al., 2017). By and large, researchers simply take the sum or the mean of the observed item scores and use this score to conduct any further analyses. An article by McNeish and Wolf (2020)—along with the entertaining exchange it spawned (McNeish, 2023; Widaman & Revelle, 2023a, 2023b)—broadly discusses shortcomings (and potential advantages) to this approach versus a latent variable approach, which excises measurement error and focuses on the true score (see also Edelsbrunner, 2022; McNeish, 2022). A more recent exchange by Sijtsma et al. (2024) and McNeish (2024) goes into further detail and provides insightful perspectives on many highly relevant issues regarding the topic at hand. It is evident that a comprehensive treatise of the topic is far beyond the scope of this singular contribution. Nonetheless, we aim to elucidate one important aspect of this topic: mean comparisons.

As per classical test theory (CTT; DeVellis, 2006), an individual’s observed test score *X* always comprises parts of true score *τ* and measurement error *ε*:

1 $$X=\tau +\varepsilon$$

and equally with regard to its variance:

2 $${\sigma }_{X}^{2}={\sigma }_{\tau }^{2}+{{\sigma }^{2}}_{\varepsilon }$$

It follows that the variance of the observed score will be greater than or equal to the variance of the true score. As a result, all calculations that are based on the observed scale score’s variance will carry a bias inversely proportional to the instrument’s reliability. This includes all calculations that incorporate measures of dispersion, such as the most common parametric statistical procedures (e.g., *t*-test, linear regression, and ANOVA). Because of its simplicity, we will use the standardized mean difference known as Cohen’s *d* to further illustrate this point (Cohen, 1992):

3 $$d=\frac{{\mu }_{1}-{\mu }_{2}}{\sigma }$$

It is evident that the pure mean difference is not affected by any changes in variance. However, any reduction (or increase) in the denominator in (3) will lead to an increase (or reduction) in the resultant effect size *d*. When bearing in mind the range of reliability parameters that are commonly deemed acceptable, this implies marked differences between the observed *d* and the true *d* that one would find with a perfect measurement. The example data in Table 1 demonstrates how, even given a constant mean difference (Δμ = 1) and constant true score variance (σ^2^_τ_ = 1), the introduction of increasing proportions of error variance leads to a misestimation of *d*.

**Table 1:** Reliability and standardized mean differences

Reliability	Error variance	Observed variance	Observed* d*
1	0	1	1
0.90	0.11	1.11	0.95
0.80	0.25	1.25	0.89
0.70	0.43	1.43	0.84
0.60	0.67	1.67	0.77
0.50	1	2	0.71

In this example, the mean difference and the true score variance were set to 1 for ease of interpretation. This results in a true *d* of 1.

As mentioned above, the measurement error component of the variance inflates the denominator in the estimation of Cohen’s *d*. The question now is what exactly this inflation signifies and whether or not it can be deemed acceptable. Per CTT, measurement error is uncorrelated to the construct of interest, and thus it seems obvious that it should be excluded from consideration (i.e., by the modeling of item-specific error terms in factor analysis). Conversely, Widaman and Revelle (2023a, 2023b) argue that the use of sum scores (and thus the inclusion of measurement error) leads to better comparability and replicability across studies. In their reasoning, that is because—in contrast to the sum score—the latent score is obtained by applying “different weightings”, i.e., the factor loadings, across studies. In our view, this statement confuses the source of differences between studies. They make it seem like the true score portion of the observed score may vary across studies. and the error portion is there to balance this out and make the sum score comparable. However, this is inaccurate. and they do not supply arguments to substantiate this claim. If we accept that the true score represents a concrete and stable construct of interest, while the error term is conceptually unrelated, random noise, then it should be evident that it is actually the error term that first introduces the lack of comparability across studies. The “differing weightings” are simply a result of differing errors (see Table 1 for an illustration of this point). Yet, importantly, if we accept that the substantial construct remains unchanged between studies, then the pattern of unstandardized loadings will also remain unchanged. But of course this construct stability should not be presumed without proper invariance testing.

The present investigation aims to examine the extent to which social sciences are affected by this issue by empirically estimating the differences between observed and latent score group differences. This is to be accomplished by analyzing a large number of openly available data sets and comparing observed and latent versions of the standardized mean difference between males and females. This comparison was chosen for the sake of feasibility, as it is the most widely available grouping variable. We expect that the latent *d* will differ significantly from the observed *d*. In addition, we expect the magnitude of this difference to be correlated with the respective scale’s reliability estimate: specifically, lower reliability should be associated with higher differences between *d*s.

## Method

### Data and participants

The data sets for the present investigation were acquired at the Open-Source Psychometrics Project (2019d) and are summarized in Table 2. We selected all questionnaires which had an ordinal response scale and only those data sets that fulfilled the conservative requirement of having at least 20 respondents per indicator. For the sake of simplicity, we included only complete observations. In total, 999,033 observations were used in the analysis.

**Table 2:** Overview of all questionnaires and samples under analysis

Questionnaire	Full name	Creator	Complete *N*
16PF	Cattell's 16 Personality Factors Test	Cattell and Mead (2008)	35,027
AS + SC + AD + DO	IPIP Assertiveness, Social confidence, Adventurousness, and Dominance scales	Golberg et al. (2006)	896
BFI-50	Big Five Personality Test	Goldberg (1992)	19,592
CFCS	Consideration of Future Consequences Scale	Strathman et al. (1994)	14,598
DASS	Depression Anxiety Stress Scales	Lovibond and Lovibond (1995)	39,156
ECR	Experiences in Close Relationships Scale	Brennan et al. (1998)	46,006
FBPS	Firstborn Personality Scale	Open-Source Psychometrics Project (2019a)	38,383
FPS	Feminist Perspectives Scale	Henley et al. (1998)	11,089
FTI	Fisher Temperament Inventory	Fisher et al. (2010)	4845
GCBS	Generic Conspiracist Beliefs Scale	Brotherton et al. (2013)	2359
GRIT	Duckworth's Grit Scale	Duckworth et al. (2007)	4165
HSNS	Hypersensitive Narcissism Scale	Hendin and Cheek (1997)	50,730
DD	The Dirty Dozen	Jonason and Webster (2010)	50,730
HSQ	Humor Styles Questionnaire	Martin et. al. (2003)	980
MACH-IV	Machiavellianism Test	Christie and Geis (1970)	72,534
MIES	Multidimensional Introversion–Extraversion Scales	Open-Source Psychometrics Project (2019b)	6893
NIS	Nonverbal Immediacy Scale	Richmond et al. (2003)	130,028
NPAS	Nerdy Personality Attributes Scale	Finister et al. (2021)	14,185
NR6	Nature Relatedness Scale	Nisbet and Zelenski (2013)	1447
OSRI44	Open Sex Role Inventory	Open-Source Psychometrics Project (2019c)	264,578
PWE	Protestant Work Ethic Scale	McHoskey (1994)	1301
RIASEC	Holland Code Test	(Holland, 1957, 1997)	134,440
RSE	Rosenberg Self-Esteem Scale	Rosenberg (1965)	45,602
RWAS	Right-wing Authoritarianism Scale	Altemeyer (1981)	9469
SCS	Sexual Compulsivity Scale	Kalichman and Rompa (1995)	3215

### Statistical analyses

All analyses were conducted using R and the packages *lavaan* and *metafor* (Rosseel, 2012; Viechtbauer, 2010). For the observed scale scores, we calculated the means/sums according to the instructions of the respective questionnaires and compared them between female and male participants using Cohen’s *d*. In our analyses, this observed standardized mean difference is called *d*_*Y*_. Analogously for the latent variable model, we first assigned items to their respective factors and fitted a multigroup confirmatory factor analysis model using robust diagonally weighted least squares estimation (*WLSMV* in *lavaan*). To enable an adequate comparison, we first constrained item thresholds, factor loadings, and item intercepts to be identical between groups (Meredith, 1993; Wu & Estabrook, 2016). It bears mentioning that this assumption is implicit in the traditional sum score approach, and thus we have to presume the same in the latent variable model in order to allow for an apples-to-apples comparison. Finally, we calculated the standardized difference between groups by directly examining the difference between latent variable means and then dividing it by the pooled standard deviation of the latent variable. This metric is called *d*_*τ*_. We then calculated the discrepancy between the two effect size estimates, Δ*d*, in both absolute terms and a signed version that retains the direction of the discrepancy. To then evaluate the difference between the two Cohen’s *d* values, we first calculated their respective variance using the formula provided by Choi and Lam (2017):

4 $${{\sigma }^{2}}_{d}=\frac{{n}_{1}+{n}_{2}}{{n}_{1}\times {n}_{2}}+ \frac{{d}^{2}}{{2(n}_{1}+{n}_{2})}$$

Based on the pooled standard deviations, we then calculated a *z* score to run a one-tailed significance test. In addition, we report the 95% confidence interval around the difference between the Cohen’s *d*s, which again uses the standard deviation of *d* based on (4). Finally, we examined the association between McDonald’s ω for each respective scale and the magnitude of the difference between the observed and latent *d*.

## Results

**Fig. 1:**
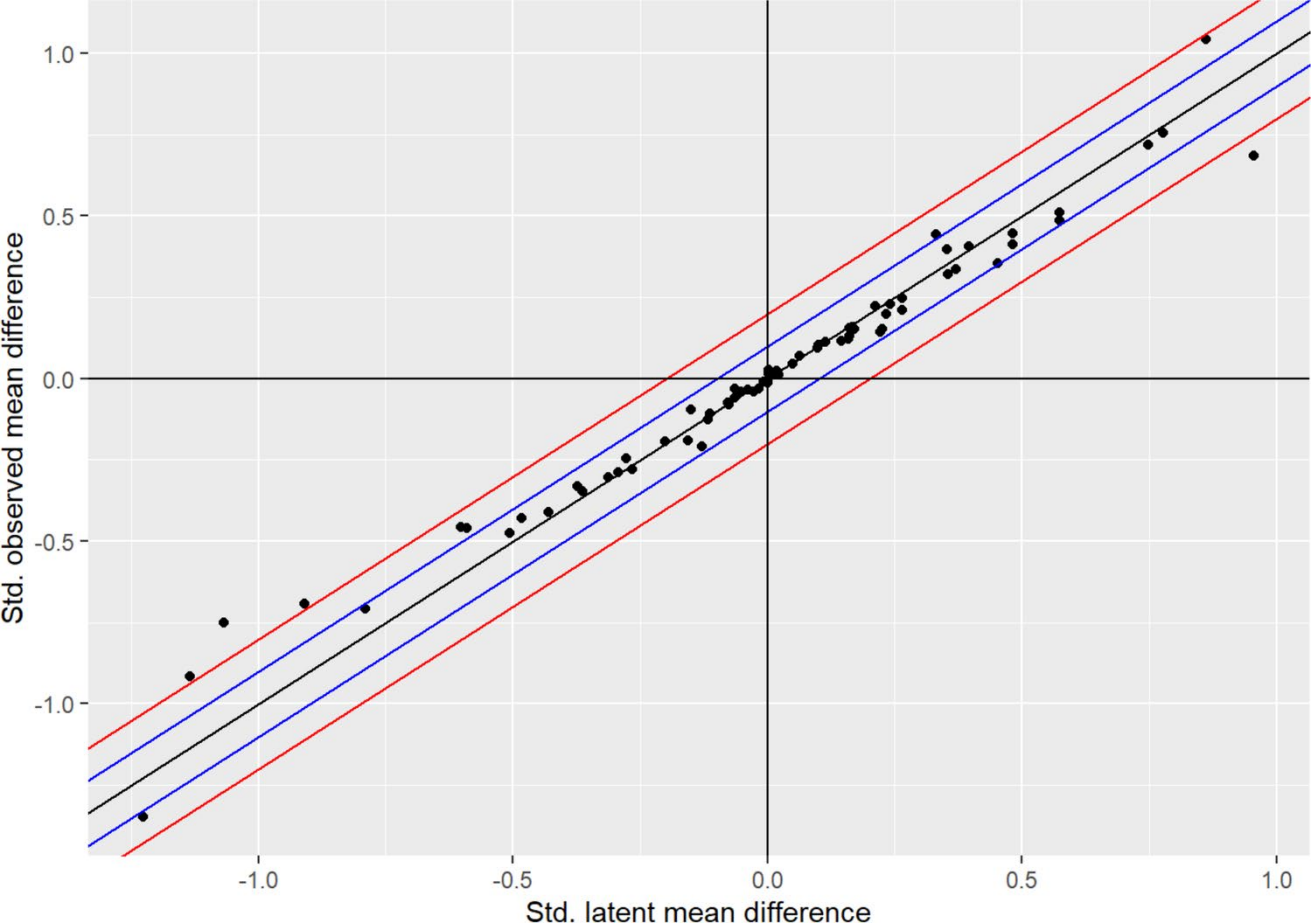
Latent mean differences versus observed mean differences. *Note.* Blue lines represent an interval of ± 0.1 *SD*; red lines represent an interval of ± 0.2 *SD*

**Fig. 2:**
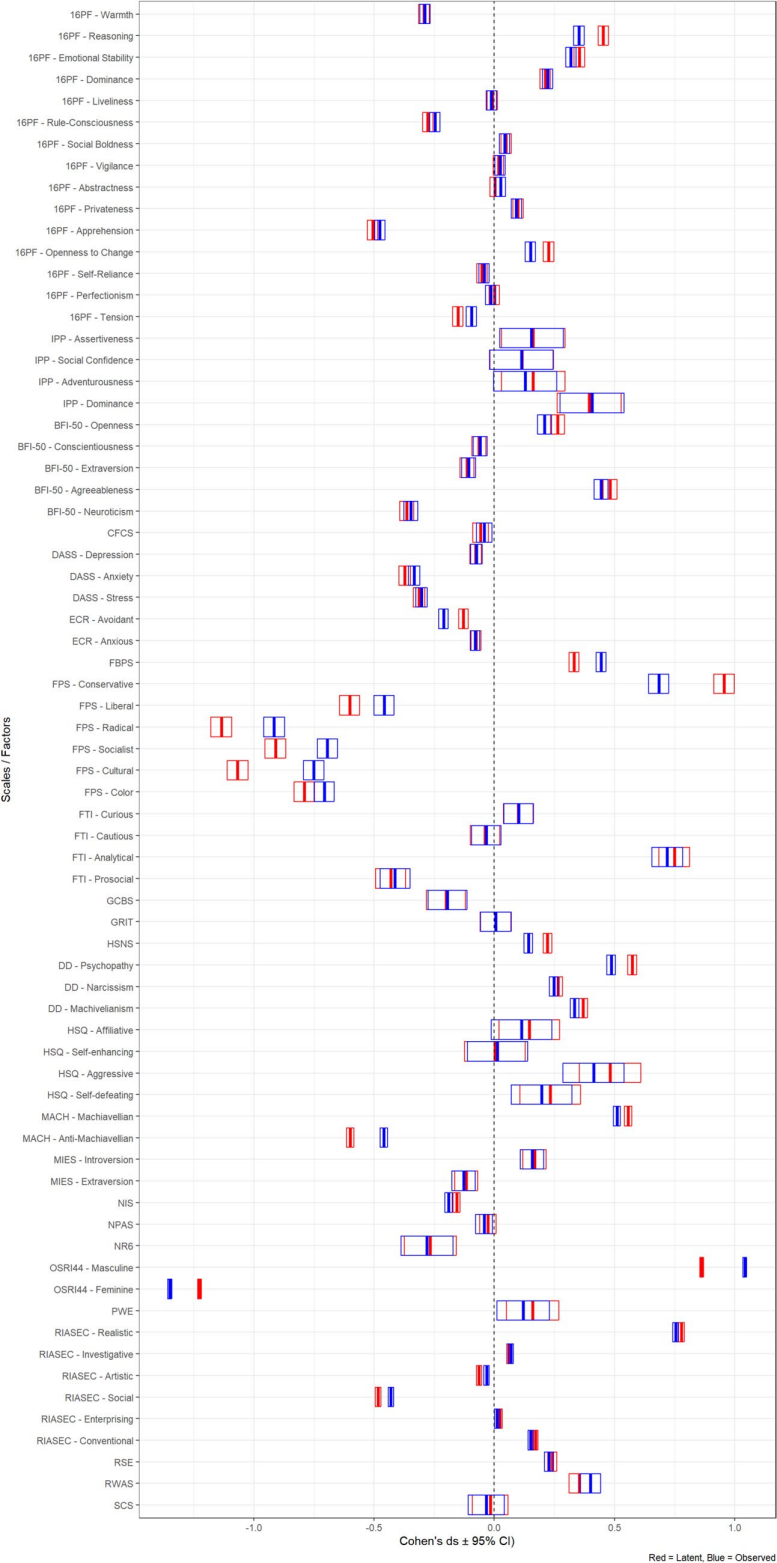
Latent and observed mean differences including 95% confidence interval

The focus of the analysis at hand was to test whether the calculation of standardized mean differences would yield significantly different results when applying a SEM framework to test the latent score difference versus simply comparing the observed scale scores. In total, we examined 71 scales. One scale (the Sensitivity subscale of Cattell’s 16PF) exhibited negative error variances and was thus omitted from the analyses. Model fit results can be found in the Supplemental Material. We summarize the findings of interest for the present study for the remaining 70 scales in Table 3. In addition, we provide a graphical representation of the results in Figs. 1 and 2. In Fig. 1, it is fairly obvious that latent and observed scores are strongly correlated but not identical, *R*^2^ = 0.970. There are some deviations from the central diagonal, which represents the expected value for cases where observed and latent mean differences are equal, but most remain within the area marked off by blue lines—representing discrepancies of less than 10% of a standard deviation. Figure 2 shows the individual comparisons in detail and includes 95% confidence intervals.

**Table 3:** Observed and latent effect size estimates and comparisons

Instrument	Subscale	${d}_{\tau }$	${d}_{Y}$	**Δ*d***	**Signed Δ*d***	**% change**	**Signed % change**	***p***	**95% CI, lower limit**	**95% CI, upper limit**	**ω**
16PF	Warmth	–0.292	–0.288	0.004	0.004	1.4	1.4	0.353	0.026	–0.035	0.872
	Reasoning	0.454	0.353	0.101	0.101	25.1	25.1	< 0.001	0.132	0.070	0.850
	Emotional stability	0.354	0.319	0.035	0.035	10.4	10.4	< 0.001	0.066	0.005	0.884
	Dominance	0.212	0.222	0.010	–0.010	4.7	–4.7	0.174	0.020	–0.041	0.860
	Liveliness	–0.008	–0.012	0.004	–0.004	43.4	–43.4	0.349	0.035	–0.026	0.846
	Rule-consciousness	–0.276	–0.247	0.030	0.030	11.3	11.3	0.003	0.001	–0.060	0.873
	Social boldness	0.049	0.044	0.006	0.006	12	12	0.305	0.036	–0.025	0.922
	Vigilance	0.018	0.025	0.007	–0.007	34.2	–34.2	0.255	0.023	–0.037	0.884
	Abstractness	0.003	0.026	0.023	–0.023	154.1	–154.1	0.019	0.008	–0.053	0.841
	Privateness	0.099	0.092	0.007	0.007	7	7	0.271	0.037	–0.024	0.896
	Apprehension	–0.506	–0.476	0.030	0.030	6.2	6.2	0.003	0	–0.061	0.872
	Openness to change	0.226	0.151	0.075	0.075	40	40	< 0.001	0.106	0.045	0.838
	Self-reliance	–0.051	–0.042	0.009	0.009	20.3	20.3	0.194	0.021	–0.040	0.888
	Perfectionism	0	–0.014	0.015	0.015	210.4	210.4	0.088	0.045	–0.015	0.823
	Tension	–0.151	–0.095	0.056	0.056	45.4	45.4	< 0.001	–0.026	–0.086	0.837
IPP	Assertiveness	0.162	0.155	0.007	0.007	4.5	4.5	0.458	0.194	–0.179	0.877
	Social confidence	0.115	0.113	0.002	0.002	1.8	1.8	0.488	0.188	–0.184	0.929
	Adventurousness	0.162	0.129	0.033	0.033	22.6	22.6	0.312	0.219	–0.153	0.870
	Dominance	0.396	0.407	0.011	–0.011	2.8	2.8	0.434	0.177	–0.199	0.901
BFI-50	Openness	0.264	0.209	0.055	0.055	23.2	23.2	< 0.001	0.096	0.014	0.861
	Conscientiousness	–0.063	–0.058	0.005	0.005	8.3	8.3	0.365	0.036	–0.046	0.836
	Extraversion	–0.113	–0.107	0.006	0.006	5.7	5.7	0.336	0.034	–0.047	0.912
	Agreeableness	0.482	0.444	0.037	0.037	8.1	8.1	0.006	0.079	–0.004	0.868
	Neuroticism	–0.363	–0.347	0.016	0.016	4.5	4.5	0.140	0.025	–0.057	0.894
CFCS		–0.057	–0.041	0.016	0.016	33.4	33.4	0.161	0.030	–0.063	0.902
DASS	Depression	–0.077	–0.073	0.004	0.004	5.3	5.3	0.372	0.030	–0.038	0.972
	Anxiety	–0.372	–0.333	0.040	0.040	11.3	11.3	< 0.001	–0.006	–0.074	0.940
	Stress	–0.312	–0.303	0.009	0.009	2.9	2.9	0.230	0.025	–0.043	0.947
ECR	Avoidant	–0.128	–0.211	0.083	–0.083	49	–49	< 0.001	0.111	0.055	0.894
	Anxious	–0.076	–0.080	0.004	–0.004	5	–5	0.349	0.032	–0.024	0.941
FBPS		0.332	0.444	0.112	–0.112	28.9	–28.9	< 0.001	–0.083	–0.141	0.821
FPS	Conservative	0.956	0.684	0.272	0.272	33.1	33.1	< 0.001	0.332	0.211	0.932
	Liberal	–0.601	–0.458	0.143	0.143	27.1	27.1	< 0.001	–0.084	–0.203	0.869
	Radical	–1.135	–0.916	0.219	0.219	21.4	21.4	< 0.001	–0.158	–0.281	0.945
	Socialist	–0.910	–0.694	0.217	0.217	27	27	< 0.001	–0.156	–0.277	0.918
	Cultural	–1.068	–0.751	0.317	0.317	34.9	34.9	< 0.001	–0.257	–0.378	0.888
	Color	–0.789	–0.707	0.082	0.082	11	11	< 0.001	–0.022	–0.142	0.918
FTI	Curious	0.100	0.101	0.002	–0.002	1.6	–1.6	0.479	0.086	–0.089	0.866
	Cautious	–0.038	–0.034	0.005	0.005	13.3	13.3	0.440	0.083	–0.092	0.840
	Analytical	0.749	0.720	0.029	0.029	4	4	0.181	0.119	–0.060	0.848
	Prosocial	–0.431	–0.413	0.018	0.018	4.3	4.3	0.283	0.070	–0.106	0.866
GCBS		–0.200	–0.195	0.005	0.005	2.8	2.8	0.447	0.109	–0.120	0.965
GRIT		0.006	0.007	0.001	–0.001	14.5	–14.5	0.488	0.089	–0.091	0.874
HSNS		0.221	0.142	0.079	0.079	43.7	43.7	< 0.001	0.105	0.054	0.806
DD	Psychopathy	0.574	0.487	0.087	0.087	16.5	16.5	< 0.001	0.113	0.061	0.808
	Narcissism	0.265	0.248	0.018	0.018	6.9	6.9	0.028	0.043	–0.008	0.830
	Machivelianism	0.370	0.334	0.036	0.036	10.1	10.1	< 0.001	0.061	0.010	0.868
HSQ	Affiliative	0.146	0.114	0.032	0.032	24.8	24.8	0.308	0.210	–0.146	0.897
	Self-enhancing	0.003	0.014	0.011	–0.011	124.9	–124.9	0.435	0.167	–0.188	0.849
	Aggressive	0.482	0.413	0.069	0.069	15.5	15.5	0.143	0.249	–0.111	0.819
	Self-defeating	0.233	0.198	0.035	0.035	16.2	16.2	0.294	0.213	–0.144	0.860
MACH	Machiavellian	0.574	0.510	0.063	0.063	8.7	8.7	< 0.001	0.084	0.042	0.856
	Anti-Machiavellian	–0.590	–0.459	0.13	0.130	26.4	26.4	< 0.001	–0.109	–0.151	0.866
MIES	Introversion	0.168	0.157	0.011	0.011	6.6	6.6	0.333	0.079	–0.058	0.944
	Extraversion	–0.117	–0.127	0.010	–0.010	8.6	–8.6	0.335	0.079	–0.058	0.949
NIS		–0.157	–0.190	0.034	–0.034	19.4	–19.4	< 0.001	0.055	0.012	0.957
NPAS		–0.026	–0.042	0.016	–0.016	46.5	–46.5	0.184	0.065	–0.033	0.900
NR6		–0.266	–0.280	0.014	–0.014	5	–5	0.403	0.167	–0.139	0.889
OSRI44	Masculine	0.863	1.042	0.180	–0.180	18.9	–18.9	< 0.001	–0.168	–0.191	0.868
	Feminine	–1.226	–1.349	0.123	–0.123	9.6	–9.6	< 0.001	0.135	0.111	0.879
PWE		0.159	0.121	0.039	0.039	27.6	27.6	0.244	0.193	–0.115	0.919
RIASEC	Realistic	0.778	0.754	0.024	0.024	3.2	3.2	< 0.001	0.041	0.008	0.902
	Investigative	0.063	0.068	0.005	–0.005	8.3	8.3	0.172	0.011	–0.021	0.931
	Artistic	–0.063	–0.032	0.031	0.031	66.2	–66.2	< 0.001	–0.015	–0.047	0.896
	Social	–0.483	–0.430	0.053	0.053	11.6	–11.6	< 0.001	–0.037	–0.069	0.894
	Enterprising	0.023	0.012	0.011	0.011	65.8	65.8	0.024	0.027	–0.005	0.881
	Conventional	0.171	0.152	0.019	0.019	11.8	11.8	< 0.001	0.035	0.003	0.921
RSE		0.242	0.227	0.014	0.014	6.1	6.1	0.071	0.041	–0.013	0.940
RWAS		0.354	0.396	0.042	–0.042	12.3	–12.3	0.027	0.018	–0.102	0.977
SCS		–0.017	–0.033	0.017	–0.017	66.4	–66.4	0.331	0.122	–0.089	0.924

*dτ* = standardized latent mean difference, *d*_*Y*_ = standardized observed mean difference, Δ*d*** = **absolute difference of *dτ* and* d*_*Y*_, % change** = **relative difference of *dτ* and* d*_*Y*_. See Table 2 for the questionnaires and meanings of the abbreviations.

Out of the 70 scales that remained in the analyses, 33 (47.1%) yielded significant differences between observed and latent *d*. There were 10 cases that exceeded a Δ*d* of 0.100 and four cases with Δ*d* > 0.200. The average absolute difference between *d*s was 0.048 (*Md* = 0.024), but had a large range of values from 0 to 0.32. The average relative change was 25.0% (*Md* = 12.0%). It should however also be mentioned that not all discrepancies were “in favor” of the latent mean difference. Twenty out of the 70 comparisons had, in absolute terms, larger observed mean differences than latent ones. Thus, when taking into account the direction of the difference, the mean difference between *d*s was 0.028 (*Md* = 0.015), ranging from –0.18 to 0.32. The average relative change was 5.9% (*Md* = 7.0%).

In order to allow for a summary assessment of the individual findings, we also conducted a meta-analysis on both the absolute and signed effect size differences. Regarding the absolute differences, the effect overall was considered significant, *d*_est_ = 0.050 [0.035, 0.066], *p* < 0.001. We found significant heterogeneity between the scales under consideration, *Q*(69) = 1144.55, *p* < 0.001, *I*^2^ = 93.98%, τ = 0.060. The results for the signed differences were similar: *d*_est_ = 0.028 [0.009, 0.046], *p* = 0.004. We again found significant heterogeneity between the scales under consideration, *Q*(69) = 2237.69, *p* < 0.001, *I*^2^ = 96.01%, τ = 0.074. In particular, the τ parameter—which represents the standard deviation of the estimated effect size—is useful for estimating the distribution of expected effect size discrepancies, because it is in the same metric as the effect size estimate itself. That is, based on these estimates, only about 1% of cases should exceed a Δ*d* of 0.200. Yet, between 5 and 16% (depending on whether one refers to absolute or signed discrepancies) of cases would exceed a Δ*d* of 0.100.

### Relation between reliability and magnitude of observed-latent effect size difference mismatch

We calculated two correlations for the associations between scale reliability as measured by ω and the magnitude of the deviation between observed and latent *d*, once in absolute terms and once for the relative (see Fig. 3.1 and 3.2). Regarding the absolute discrepancy, there was a negligible association with ω, *r*(68) = –0.026 [–0.259, 0.210], *p* = 0.833. With regard to the percentage change, however, the association with ω was significant, *r*(68) = –0.290 [–0.492, –0.059], *p* = 0.015. This means that the higher a given scale’s internal consistency, the smaller the disconnect between observed and latent mean group differences—in relative terms. However, it should be noted that the relationship for the relative change parameter appears to be driven by a small number of extreme data points. Accordingly, when redoing the analysis using Spearman rank correlation, it is smaller and nonsignificant, *ρ*(68) = –0.227 [–0.467, 0.012], *p* = 0.059.

**Fig. 3:**
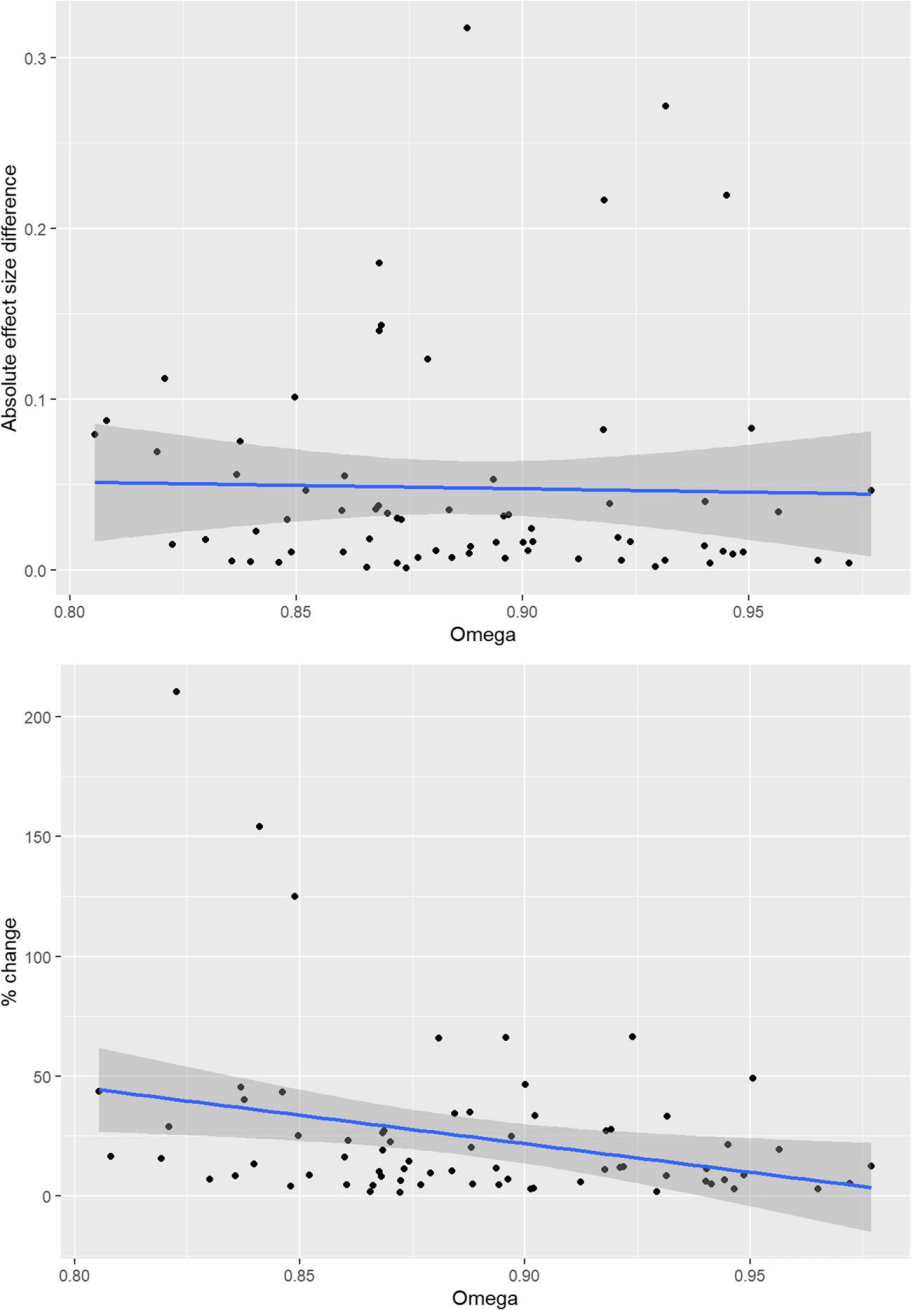
**3.1** The relationship between absolute mean difference discrepancy and internal consistency as measured by ω.** 3.2** The relationship between percent change mean difference discrepancy and internal consistency as measured by ω

## Discussion

The present study sought to investigate two different scale scoring methods, that is, scoring by summing/averaging (observed score) versus the more elaborate latent variable approach (latent score). Specifically, we were interested in quantifying the extent to which the two do in fact differ and whether it is practically relevant and necessary to differentiate between the two in most research questions, or whether they arrive at the same conclusion anyways. As Widaman and Revelle (2023b) aptly put it, the “proof is in the pudding, not in a priori declaration by fiat.” Our findings based on the analysis of an extensive personality research database show that simple group comparison results can vary significantly based on the employed scale scoring method. It should be noted that the samples we considered in the present investigation were generally large, and thus significant results are easily achieved even with small effect sizes. Further, it bears mentioning that the average difference between Cohen’s *d* estimates was indeed small. However, it should not be neglected in relative terms. Moreover, there was substantial variation across scales with regard to the extent of this bias. Since a lower reliability equals a larger proportion of error variance, with the removal of the same we expected a larger impact on the standardized mean difference. We were, however, unable to show this in the present study. This may be due to the generally high level of scale reliability (average ω was 0.89, the lowest was still 0.81). Thus, future research should consider tackling this question again since it can be shown quite easily analytically that low reliability scales will have higher discrepancies between observed and latent mean differences.

Our findings line up well with the potential issues with sum scoring raised by McNeish and Wolf (2020) in their initial contribution on the topic. In particular, they pointed out that inappropriate use of the sum scoring method puts into question a scale’s reliability and validity. McNeish and Wolf chiefly emphasize the impact on individual scoring (as opposed to the group-level means, which we considered in this study). While Widaman and Revelle (2023a) correctly point out that the models under study exhibited unacceptable fit, this does in our view not fully alleviate the issues of discrepancies between observed scores and factor-based scores (or in our case, latent variable means). Instead, it serves to emphasize that given high measurement quality and good model fit, the simple sum score can typically be expected to adequately approximate factor scores (or latent variable means) obtained in confirmatory factor analysis. Finally, McNeish (2023) shows in various simulations that even given near-1 correlations of observed and latent scores, the utilization of sum scores can lead to inappropriate score classifications as well as decreased reliability. In the present study, we similarly found very high correlations between observed and latent mean differences across scales. Nonetheless, about half of the presented scales deviated significantly between observed and latent mean group comparisons. The significant association of the reliability estimate with the mean difference discrepancy is of particular interest.

In practical terms, the present research does *not* suggest that any and all mean comparisons on scales definitely need to be carried out using a latent variable approach. Both the observed mean difference and the latent mean difference may have their place and time in various research questions. If only one thing were to be taken away from this research, it is the need for reliable measures, as these minimize the impact of measurement error on group comparisons. Nonetheless, given the imperfection of all measurements of latent constructs, studies should strongly consider employing factor analysis if possible within the confines of their respective research question and design. Counter-arguments against using factor analysis could be small sample size and potential issues with model specification. However, especially in such cases where strong intergroup differences are expected, it becomes paramount to reliably estimate them, since the bias will—in absolute terms—be stronger here. As a rule of thumb, we suggest that any scales with reliability estimates of 0.80 or lower should strongly consider using a latent variable approach, since in such cases the bias in the standardized mean difference is expected to exceed 10%.

### Limitations and future research

In the present study, we found some unexpected results where the magnitude of the observed mean difference actually exceeded the latent mean difference. This result is surprising, because the excision of error variance should generally lead to a reduction in the denominator portion of the standardized mean difference and thus an increase in effect size magnitude (e.g., ECR, FBPS, and OSRI). It should however be noted that scale reliability is not the only factor affecting the denominator variance and, as a result, the evaluation of standardized mean differences. Future research should consider investigating these exceptions and what caused them.

In particular, the CTT assumption of uncorrelated errors, which is essential for traditional latent variable models in SEM, bears mentioning. This assumption may be violated in some cases, as evidenced by significant clusters of correlated error terms in one’s item subsets. In the authors’ view, this would indicate that portions of (at least partially) construct-related variance have been constrained to the error terms despite them being construct-related to some extent. Thus, the factor analytical approach for testing mean differences may in fact be too liberal in such cases.

Furthermore, in many cases, measurement invariance (or rather a lack thereof) and its conceptual twin, differential item and test function, play an important role in explaining group differences in scale usage and interpretation. In terms of the mean structure, however, lack of scalar invariance does not per se change the obtained latent variable means. It does, however, call into question the validity of inferences made with regard to score-level group differences. It should however be emphasized that the same is true for observed score comparisons. The present investigation disregards this issue in order to focus exclusively on the role of reliability (or measurement error) in conducting group comparisons.

Finally, there are some phenomena that are not directly related to the construct of interest but can still systematically affect the measurement process, such as social desirability and acquiescence (Podsakoff et al., 2003). There is a plethora of research on how these can affect estimates of differences and associations and as many ways to remedy these issues. Thus, we will omit a detailed discussion of the topic at this point.

## Conclusion

In sum, the present investigation demonstrated that one can expect significant differences between the results of latent and observed mean comparisons, but generally the magnitude of the effect size is rather small with few exceptions. We discussed factors that impact the magnitude of this bias as well as practical implications. We want to emphasize the importance of carefully selecting reliable and standardized scales, as well as choosing appropriate scale scoring methods, in order obtain correct and comparable study results.

## Supplementary Information

Below is the link to the electronic supplementary material.

Supplementary file1 (DOCX 26 KB)

## Data Availability

All data sets are openly available at https://openpsychometrics.org/_rawdata.

## Code availability

All code is available at https://github.com/bschmalbach/ObsLatMeans.
