# Supplementary material for: What you see is not what you get: Observed scale score comparisons misestimate true group differences
Source: Behav Res Methods. 2025 Mar 19;57(4):122. doi: 10.3758/s13428-025-02639-w (PMC11923020; doi:10.3758/s13428-025-02639-w)
Supplement: Supplementary file 1 — Supplementary file1 (DOCX 26 KB) [file 13428_2025_2639_MOESM1_ESM.docx]

Table S1
*Model fit results*

| Scales | χ² | *df* | *p* | *CFI* | *TLI* | *RMSEA* | *SRMR* |
| --- | --- | --- | --- | --- | --- | --- | --- |
| 16PF - Warmth | 13674.24 | 108 | <.001 | .938 | .948 | .085 | .059 |
| 16PF - Reasoning | 27512.26 | 180 | <.001 | .847 | .867 | .093 | .078 |
| 16PF - Emotional Stability | 15421.61 | 108 | <.001 | .943 | .953 | .090 | .055 |
| 16PF - Dominance | 20212.41 | 108 | <.001 | .922 | .935 | .103 | .068 |
| 16PF - Liveliness | 40027.01 | 108 | <.001 | .843 | .869 | .145 | .122 |
| 16PF - Rule-Consciousness | 19780.87 | 108 | <.001 | .946 | .955 | .102 | .077 |
| 16PF - Social Boldness | 12378.07 | 108 | <.001 | .975 | .979 | .081 | .042 |
| 16PF - Vigilance | 17195.55 | 108 | <.001 | .945 | .954 | .095 | .061 |
| 16PF - Abstractness | 50044.33 | 108 | <.001 | .852 | .877 | .162 | .129 |
| 16PF - Privateness | 13724.97 | 108 | <.001 | .970 | .975 | .085 | .049 |
| 16PF - Apprehension | 10972.86 | 108 | <.001 | .951 | .959 | .076 | .046 |
| 16PF - Openness to Change | 15562.76 | 108 | <.001 | .916 | .930 | .090 | .073 |
| 16PF - Self-Reliance | 19991.82 | 108 | <.001 | .928 | .940 | .103 | .070 |
| 16PF - Perfectionism | 36835.53 | 108 | <.001 | .797 | .830 | .139 | .110 |
| 16PF - Tension | 23104.5 | 108 | <.001 | .915 | .930 | .110 | .077 |
| IPP - Assertiveness | 453.7887 | 108 | <.001 | .948 | .957 | .085 | .070 |
| IPP - Social Confidence | 1233.271 | 108 | <.001 | .914 | .928 | .153 | .092 |
| IPP - Adventurousness | 859.5205 | 108 | <.001 | .911 | .926 | .125 | .102 |
| IPP - Dominance | 1774.903 | 108 | <.001 | .832 | .86 | .186 | .146 |
| BFI-50 - Openness | 22535.14 | 108 | <.001 | .822 | .852 | .146 | .111 |
| BFI-50 - Conscientiousness | 7337.383 | 108 | <.001 | .925 | .938 | .083 | .062 |
| BFI-50 - Extraversion | 10443.14 | 108 | <.001 | .952 | .960 | .099 | .052 |
| BFI-50 - Agreeableness | 7326.253 | 108 | <.001 | .944 | .953 | .083 | .059 |
| BFI-50 - Neuroticism | 15838.01 | 108 | <.001 | .929 | .940 | .122 | .075 |
| CFCS | 5466.557 | 154 | <.001 | .960 | .965 | .069 | .050 |
| DASS - Depression | 42844.42 | 194 | <.001 | .972 | .974 | .106 | .043 |
| DASS - Anxiety | 23693.67 | 194 | <.001 | .958 | .961 | .079 | .050 |
| DASS - Stress | 29747.02 | 194 | <.001 | .957 | .960 | .088 | .045 |
| ECR - Avoidant | 130992.4 | 340 | <.001 | .894 | .904 | .129 | .075 |
| ECR - Anxious | 137065.3 | 340 | <.001 | .836 | .852 | .132 | .084 |
| FBPS | 98475.76 | 700 | <.001 | .799 | .814 | .085 | .074 |
| FPS - Conservative | 1338.507 | 108 | <.001 | .979 | .983 | .045 | .044 |
| FPS - Liberal | 7133.819 | 108 | <.001 | .781 | .817 | .108 | .108 |
| FPS - Radical | 3386.817 | 108 | <.001 | .978 | .982 | .074 | .044 |
| FPS - Socialist | 2310.31 | 108 | <.001 | .976 | .980 | .061 | .025 |
| FPS - Cultural | 3167.202 | 108 | <.001 | .934 | .945 | .071 | .048 |
| FPS - Color | 3200.931 | 108 | <.001 | .973 | .977 | .072 | .039 |
| FTI - Curious | 4944.8 | 194 | <.001 | .845 | .855 | .101 | .084 |
| FTI - Cautious | 3807.607 | 194 | <.001 | .880 | .887 | .088 | .079 |
| FTI - Analytical | 8326.393 | 194 | <.001 | .714 | .732 | .132 | .108 |
| FTI - Prosocial | 11253.02 | 194 | <.001 | .763 | .778 | .153 | .148 |
| GCBS | 5277.373 | 238 | <.001 | .924 | .933 | .134 | .093 |
| GRIT | 4984.932 | 156 | <.001 | .847 | .871 | .122 | .108 |
| HSNS | 80992.35 | 108 | <.001 | .634 | .695 | .171 | .132 |
| DD - Psychopathy | 1111.881 | 18 | <.001 | .992 | .995 | .049 | .032 |
| DD - Narcissism | 4348.358 | 18 | <.001 | .972 | .981 | .097 | .032 |
| DD - Machivelianism | 1471.052 | 18 | <.001 | .994 | .996 | .056 | .018 |
| HSQ - Affiliative | 285.5775 | 70 | <.001 | .969 | .975 | .079 | .056 |
| HSQ - Self-enhancing | 325.6203 | 70 | <.001 | .953 | .962 | .086 | .069 |
| HSQ - Aggressive | 253.1706 | 70 | <.001 | .944 | .955 | .073 | .060 |
| HSQ - Self-defeating | 185.4199 | 70 | <.001 | .982 | .986 | .058 | .045 |
| MACH - Machiavellian | 542.5817 | 108 | <.001 | .982 | .985 | .040 | .036 |
| MACH - Anti-Machiavellian | 1264.869 | 108 | <.001 | .956 | .963 | .065 | .051 |
| MIES - Introversion | 52611.05 | 2068 | <.001 | .757 | .767 | .084 | .078 |
| MIES - Extraversion | 46127.98 | 2160 | <.001 | .764 | .774 | .077 | .072 |
| NIS | 1446085 | 700 | <.001 | .705 | .726 | .178 | .161 |
| NPAS | 75529.24 | 726 | <.001 | .430 | .490 | .121 | .107 |
| NR6 | 182.9672 | 40 | <.001 | .979 | .985 | .070 | .047 |
| OSRI44 - Masculine | 668605.4 | 504 | <.001 | .980 | .982 | .100 | .091 |
| OSRI44 - Feminine | 338541.1 | 504 | <.001 | .805 | .822 | .071 | .062 |
| PWE | 1868.619 | 378 | <.001 | .919 | .927 | .078 | .069 |
| RIASEC - Realistic | 46509.42 | 70 | <.001 | .957 | .965 | .099 | .056 |
| RIASEC - Investigative | 125390.7 | 70 | <.001 | .938 | .950 | .163 | .086 |
| RIASEC - Artistic | 68705.71 | 70 | <.001 | .938 | .951 | .121 | .063 |
| RIASEC - Social | 55921.13 | 70 | <.001 | .937 | .950 | .109 | .060 |
| RIASEC - Enterprising | 62898.33 | 70 | <.001 | .917 | .934 | .116 | .053 |
| RIASEC - Conventional | 37715.75 | 70 | <.001 | .976 | .981 | .089 | .035 |
| RSE | 40318.5 | 98 | <.001 | .953 | .957 | .134 | .062 |
| RWAS | 22878.03 | 592 | <.001 | .949 | .960 | .089 | .056 |
| SCS | 1791.499 | 98 | <.001 | .957 | .961 | .104 | .059 |
